# Overexpression of Active Aurora-C Kinase Results in Cell Transformation and Tumour Formation

**DOI:** 10.1371/journal.pone.0026512

**Published:** 2011-10-27

**Authors:** Jabbar Khan, Frédéric Ezan, Jean-Yves Crémet, Alain Fautrel, David Gilot, Marine Lambert, Christelle Benaud, Marie-Bérengère Troadec, Claude Prigent

**Affiliations:** 1 CNRS, UMR 6061, Institut Génétique et Développement de Rennes, Rennes, France; 2 Université Rennes 1, UEB, IFR 140, Faculté de Médecine, Rennes, France; 3 IRSET, EA 4427-SeRAIC, Rennes, France; 4 Histopathology Platform H2H2, IFR140, Biogenouest, Rennes, France; 5 INSERM U991, Rennes, France; 6 MRic Microscopy Facility, IFR140, Rennes, France; Institut de Génomique Fonctionnelle de Lyon, France

## Abstract

Aurora kinases belong to a conserved family of serine/threonine kinases key regulators of cell cycle progression. Aurora-A and Aurora-B are expressed in somatic cells and involved mainly in mitosis while Aurora-C is expressed during spermatogenesis and oogenesis and is involved in meiosis. Aurora-C is hardly detectable in normal somatic cells. However all three kinases are overexpressed in many cancer lines. Aurora-A possesses an oncogenic activity while Aurora-B does not. Here we investigated whether Aurora-C possesses such an oncogenic activity. We report that overexpression of Aurora-C induces abnormal cell division resulting in centrosome amplification and multinucleation in both transiently transfected cells and in stable cell lines. Only stable NIH3T3 cell clones overexpressing active Aurora-C formed foci of colonies when grown on soft agar, indicating that a gain of Aurora-C activity is sufficient to transform cells. Furthermore, we reported that NIH-3T3 stable cell lines overexpressing Aurora-C induced tumour formation when injected into nude mice, demonstrating the oncogenic activity of enzymatically active Aurora kinase C. Interestingly enough tumor aggressiveness was positively correlated with the quantity of active kinase, making Aurora-C a potential anti-cancer therapeutic target.

## Introduction

Aurora kinases belong to a conserved family of serine/threonine kinases that are pivotal to the successful execution of cell division. Three Aurora kinases (Aurora-A, -B, and –C), which share sequence homology in their central catalytic kinase domains, have been identified in mammals [1]. Yeast genome encodes only one member-IpL1 of this kinase family, but there are two members of this family in Drosophila. The three members of the mammalian family, besides being implicated as mitotic regulators, have generated significant interest in the cancer research field due to their elevated expression profiles detected in many human cancers [2]–[5].

Aurora-A is ubiquitously expressed especially in tissues with high mitotic and meiotic index. Aurora-A mRNA, protein expression levels and kinase activity are cell cycle regulated, low in G1/S phase, peaking in G2/M and then dropping upon mitotic exit into the next G1 [6], [7]. Aurora-A displays dynamic subcellular localization: from duplicated centrosomes at the end of S phase to mitotic spindle poles from prophase through telophase. Activation of centrosomal Aurora-A at late G2 phase is essential for centrosome maturation and mitotic entry. Its further activation are required for centrosome separation, leading to subsequent bipolar spindle formation and chromosomal alignment. Aurora-A is found overexpressed in a large number of tumours [8]–[16]. Aurora-A is an oncogene. It induces tumour formation when NIH-3T3 or Rat1 cells overexpressing Aurora-A are injected in nude mice [11]–[13], [17]–[18].

Aurora-B expression peaks at G2/M phase and the maximum kinase activity is reached at transition during metaphase to anaphase [19]. Aurora-B is responsible for histone H3 phosphorylation on both Ser-10 and Ser-28 during mitosis [6], [20]. Aurora-B is also required to correct syntelic attachments of chromosomes [7] and is essential for cytokinesis.

Unlike Aurora-A and -B, which are ubiquitously expressed in many tissues, particularly in mitotically dividing cells, Aurora-C is predominantly expressed in the testis [21], [22] and in meiotically dividing gametes where it is associated with INCENP in spermatocytes [23], [24]. Aurora-C is, however, found at a low level in other tissues [25]. Aurora-C directly competes with Aurora-B for binding to INCENP and survivin [5], [26]. Overexpression of Aurora-C in cancerous tissues and cell lines also raises questions about its potential role in carcinogenesis and its effect on the proliferative capacity of tumour cells [27], [28].

Here we asked whether Aurora kinase C has any oncogene activity. We found that Aurora kinase C causes both centrosome amplification and multinucleation and also has the capability to transform NIH-3T3 cells when overexpressed. Furthermore, we show that NIH-3T3 cells overexpressing Aurora kinase C promote tumour formation when injected into nude mice. Hence, we provide evidence that Aurora-C is a proto-oncogene.

## Results

We address the issue of the implication of Aurora-C (aurC) in cancer. Our goal was to determine if Aurora-C was an oncogene when overexpressed in somatic cells. To achieve that purpose, we overexpressed human Aurora-C tagged with GFP in mouse NIH-3T3 cells. We studied in these cells ploidy and centrosome number, the capability of transformation when grown in soft agar, and the induction of tumour when injected into immunocompromised mice.

### GFP-AuroraC-wild type and GFP-AuroraC-T191D are active kinase

**Figure 1:**
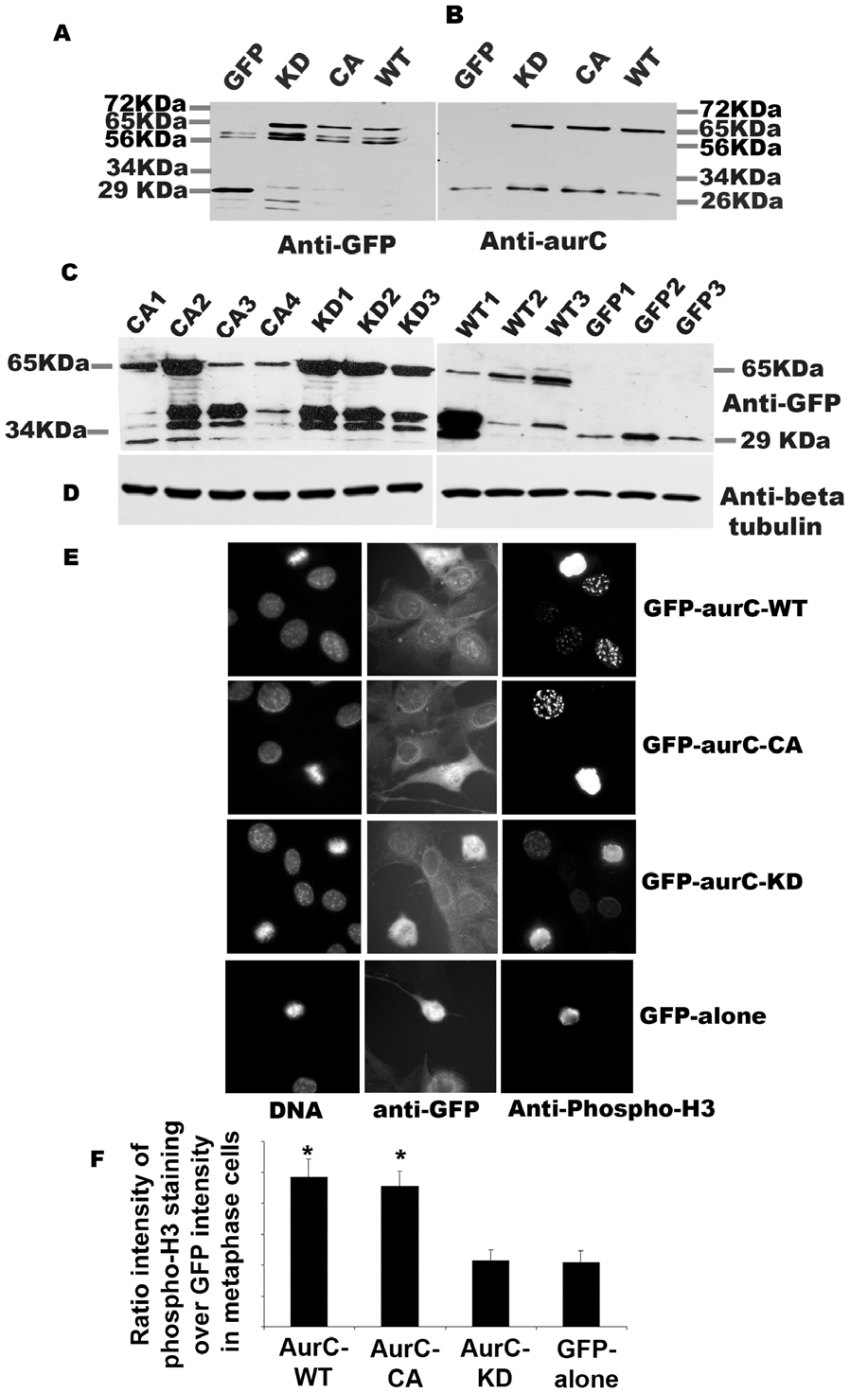
Expression and activity of GFP-Aurora-C in transiently transfected cells and in stable cell lines. (**A–B**) NIH-3T3 cells were transfected with GFP-aurC-WT (Aurora-C-wild type), GFP-aurC-CA (Aurora-C-T191D, constitutively active), GFP-aurC-KD (Aurora-C-K72R, kinase dead) and GFP-alone plasmids. Proteins were extracted 24 hours after transfection and immunoprecipitated with mouse anti-GFP (A) and polyclonal rabbit anti-Aurora-C (B) antibodies. Western blots were revealed using (A) mouse Anti-GFP antibody and (B) polyclonal rabbit Anti-Aurora-C antibody and showed level of expression of GFP-aurC at about 65 kDa and GFP-alone at 29 kDa. (**C–D**) Western blots showed the level of expression of GFP-aurC protein in stable NIH-3T3 clones of GFP-aurC-KD (KD1 to KD3), GFP-aurC-CA (CA1 to CA4), GFP-aurC-WT (WT1 to WT3) and GFP-alone (GFP1 to GFP3) illustrating the different level of expression of GFP-aurC and GFP proteins by different clones. The antibodies were (**C**) mouse anti-GFP and (**D**) anti-β tubulin antibody for loading control. (**E**) Immunofluorescence was performed on stable cell clones overexpressing GFP-aurC-WT, GFP-aurC-CA, GFP-aurC-KD and GFP-alone plasmids. Cells were stained with rabbit anti-phospho histone H3 ser-10 then anti-rabbit Alexa-555 and DAPI. The left column shows DAPI stained cells and the right column shows phosphorylated Histone-H3 ser-10 cells. (F) Histogram shows the ratio of H3-staining intensity (arbitrary unit from ImageJ) over GFP intensity in metaphase cells. A minimum of 300 cells were counted for each condition. We performed non-parametric Mann-Whitney test. Results were considered as statistically significant (*) for a p-value under 0.05 when compared to GFP-alone(*) condition.

We transiently transfected NIH-3T3 cells with GFP-aurC-WT (wild type), GFP-aurC-CA (constitutively active, T191D), GFP-aurC-KD (kinase dead, K72R) and GFP-alone (negative control vector) plasmids. We controlled the expression of GFP-aurC proteins 24 hours after transfection by Western blot with two different antibodies, anti-GFP and anti-Aurora-C **(** **Figure 1A, B** **)**. We clearly identified GFP-aurC in GFP-aurC-WT, GFP-aurC-CA and GFP-aurC-KD at 65KDa with anti-GFP and anti-Aurora-C antibodies. This band was not present in GFP-alone samples. The blot with anti-GFP showed a non-specific band at about 56 KDa and the blot with polyclonal anti-aurC antibody showed also a non-specific band of 26 KDa. We never detected any endogenous Aurora-C in NIH-3T3 cells by Western blot. Like other Aurora kinases, Aurora kinase C phosphorylates Histone H3 at serine-10 [26], [29]–[32]. *In vitro* kinase assays using histone H3 as a substrate confirmed the activity of immunoprecipitated GFP-aurC-WT and GFP-aurC-CA, and the inactivity of GFP-aurC-KD and GFP-alone proteins (data not shown).

We also generated stable cell lines with GFP-aurC-WT, GFP-aurC-CA, GFP-aurC-KD and GFP-alone. We checked the level of expression of GFP-aurC and GFP-alone proteins in all stable cell clones with anti-GFP antibody **(** **Figure 1 C, D** **)**.

We checked *in vivo* kinase activity of GFP-aurC-WT, GFP-aurC-CA, GFP-aurC-KD and GFP-alone in stable cell lines and quantified the level of phosphorylation of Histone H3 by immunofluorescence relative to mitotic cells. Intensity of phosphorylation of Histone H3-serine-10 was found two fold higher with GFP-aurC-WT or GFP-aurC-CA compared to GFP-aurC-KD or GFP-alone **(** **Figure 1E, F** **)**.

We concluded that GFP-AurC-WT and GFP-aurC-CA were active kinases *in vivo* while GFP-aurC-KD was inactive.

### Overexpressed GFP-Aurora kinase C localizes like a chromosomal passenger protein

**Figure 2:**
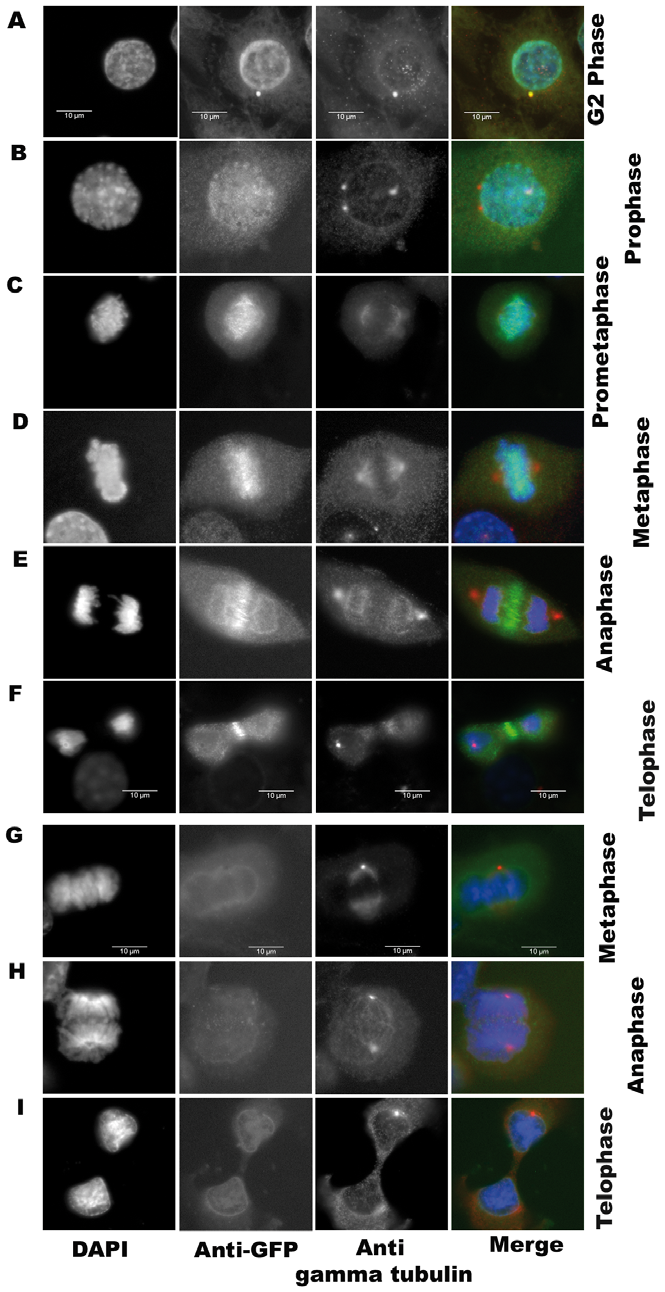
Subcellular localization of GFP-Aurora C along cell cycle. Immunofluorescence was performed on stable cell clones overexpressing GFP-aurC-WT, GFP-aurC-CA, GFP-aurC-KD and GFP-alone plasmids. Cells were stained with DAPI, anti-GFP and anti-gamma-tubulin antibodies. The immunoflorescent microscopy images show the localization of (**A-F**) GFP-aurC-WT and (**G-I**) GFP-alone. Localization of GFP-aurC-WT at (**A**) centrosome in G2 phase, (**B**) chromosomes/centromeres in prophase, (**C**) chromosomes in prometaphase, (**D**) chromosomes in metaphase, (**E**) the midzone of spindles in anaphase, (**F**) midbody in telophase. (**G-I**) Localization of GFP-alone in (**G**) metaphase, (**H**) anaphase, and (**I**) telophase. The original magnification used was 63×.

**Figure 3:**
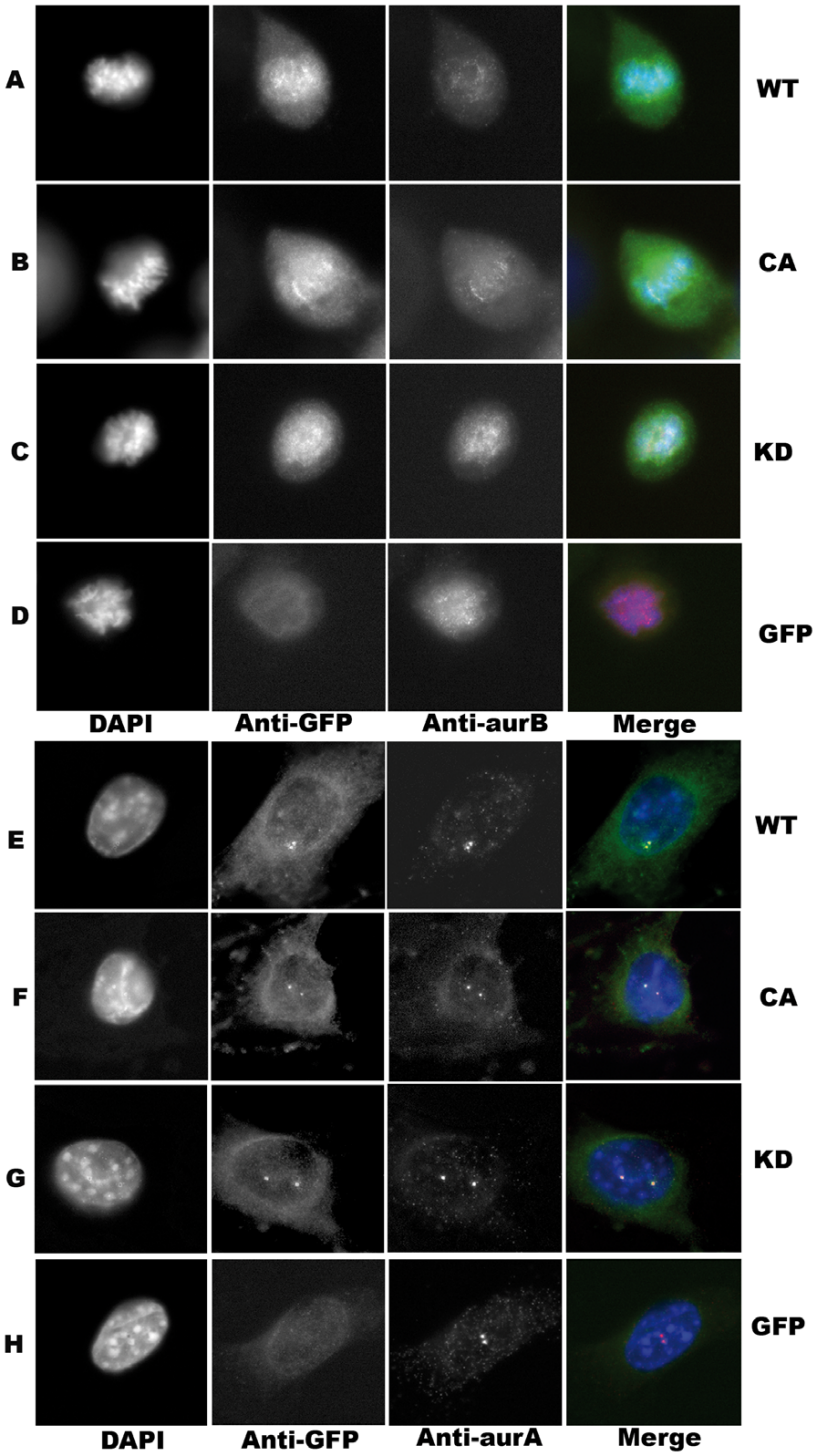
Localization of endogenous Aurora-A and Aurora-B in cell line overexpressing GFP-aurC. Immunofluorescence was performed on stable cell clones overexpressing GFP-aurC-WT, GFP-aurC-CA, GFP-aurC-KD and GFP-alone plasmids. Cells were stained with DAPI, anti-GFP, and anti-Aurora-B antibodies (**A-D**) or anti-Aurora-A antibodies (**E-H**). The immunoflorescent microscopy images show also the localization of (**A, E**) GFP-aurC-WT, (**B, F**) GFP-aurC-CA, (**C, G**) GFP-aurC-KD and (**D, H**) GFP-alone during metaphase (**A-D**) or interphase (**E-F**). The original magnification used was 63×.

We first checked by immunofluorescence whether the GFP tag perturbed the localization of overexpressed Aurora-C along the cell cycle. As already reported AurC localizes at centrosome in G2, on chromosomes from prophase to metaphase and at the midbody from anaphase to telophase [33]. As expected we found GFP-aurC-WT, GFP-aurC-CA and GFP-aurC-KD localized on duplicated centrosomes in G2 phase, like Aurora-A **(** **Figure 2A** and data not shown) where it may interfere with Aurora-A functions as expected [19] [33]. Overexpression of GFP-AurC (GFP-aurC-WT, GFP-aurC-CA and GFP-aurC-KD) does not displace endogenous Aurora-A out of the centrosomes (Figure 3E–H).

During mitosis, GFP-aurC-WT, GFP-aurC-CA and GFP-aurC-KD localized at kinetochores of the chromosomes during prophase and metaphase. They relocalized at the midzone of spindles during anaphase and at the midbody during telophase **(** **Figure 2B–F** and data not shown). The GFP tag does not perturb the localization of overexpressed Aurora-C. All three forms of GFP-aurC (WT, CA and KD) are localized as expected. Overexpression of active GFP-AurC (GFP-aurC-WT and GFP-aurC-CA) displaces endogenous Aurora-B (Figure 3A–D) and decreases Aurora-B protein level. Overexpression of GFP-AurC-KD does not displace endogenous Aurora-B. Thus, overexpressed Aurora-C is a mitotic chromosome passenger protein that behaves like Aurora-B and may interfere with its functions during mitosis [5], [26], [33].

### Overexpression of active Aurora-C induces mitotic defects: centrosome amplification and multinucleation

**Figure 4:**
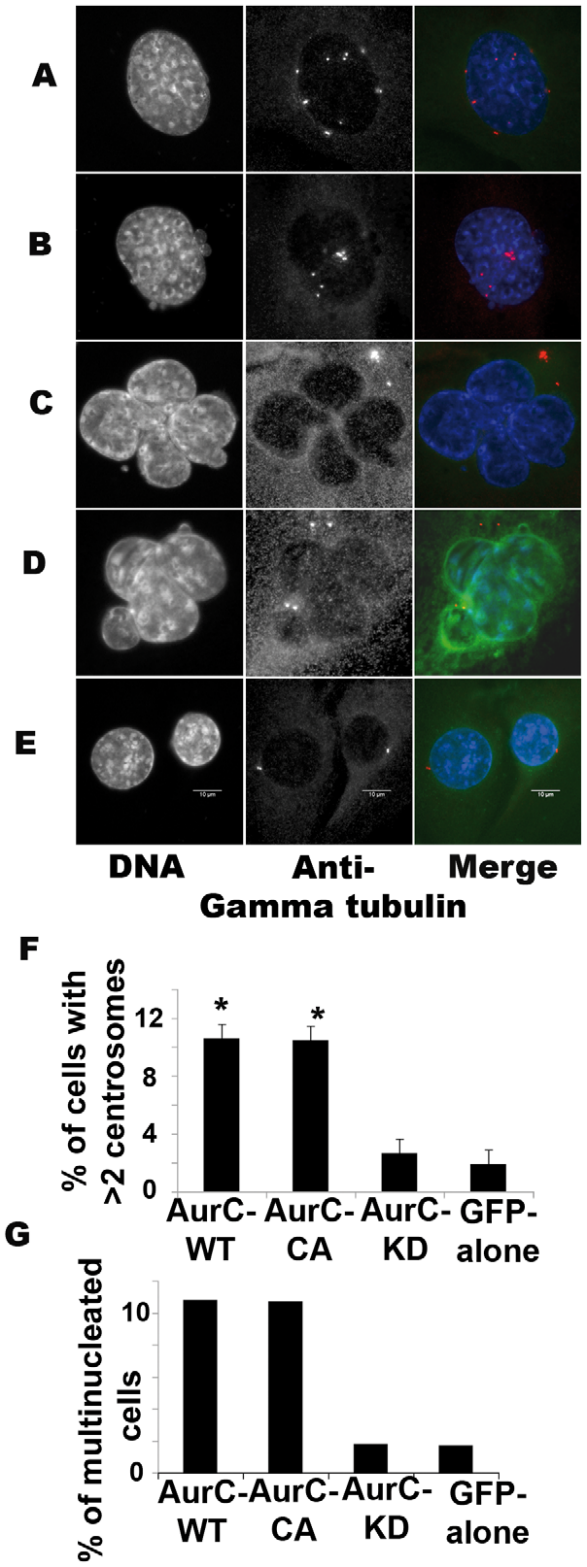
Centrosome number and multinucleation. NIH-3T3 cells were transfected with (**A, C**) GFP-aurC-WT, (**B, D**) GFP-aurC-CA and (**E**) GFP-aurC-KD vectors, fixed after 96 hours and stained with DAPI and Anti-γ tubulin antibody. (**A-B**) More than two centrosomes/cell appeared as white dots with anti-γ tubulin staining in (**A**) GFP-aurC-WT and (**B**) GFP-aurC-CA transfected cells. (**C-D**) Multinucleation (more than one nucleus/cell) in (**C**) GFP-aurC-WT and (**D**) GFP-aurC-CA in transfected cells. (**E**) Two centrosomes per cell and only one nucleus/cell in G2 phase of GFP-aurC-KD transfected cells. (**F**) Histogram shows the percentage of cells with more than 2 centrosomes/cell of 96 hours GFP-aurC-WT, GFP-aurC-CA, GFP-aurC-KD and GFP-alone transfected cells. (**G**) Histogram shows the percentages of multinucleated cells of 96 hours GFP-aurC-WT, GFP-aurC-CA, GFP-aurC-KD and GFP-alone transfected cells. A minimum of 600 cells was counted for each condition. We performed non-parametric Mann-Whitney test. Results were considered as statistically significant (*) for a p-value under 0.05 when compared to GFP-alone condition.

We used gamma-tubulin staining, a centrosomal marker, to assess centrosome number, and DNA staining to assess multinucleation (more than one nucleus per cell) by immunofluorescence. The percentage of cells with more than 2 centrosomes per cell expressing GFP-aurC-WT or GFP-aurC-CA was 5 times higher compared to GFP-aurC-KD or GFP-alone in transiently transfected NIH-3T3 cells **(** **Figure 4A, B, E, F** **)**, in NIH-3T3 stable cell lines (data not shown) and in NIH-3T3 cells with similar vectors lacking GFP-fusion protein (data not shown). We also found that the percentage of multinucleated cells was 5 times higher with GFP-aurC-WT or GFP-aurC-CA compared to GFP-aurC-KD or GFP-alone in transiently transfected NIH-3T3 cells **(** **Figure 4C–E, G** **)** and in the stable cell lines (data not shown). We finally extracted nuclei from GFP-aurC-WT, GFP-aurC-CA, GFP-aurC-KD and GFP-alone cells (n = 4 each). These nuclei were stained with propidium iodide and analysed by flow cytometry as in [34]. We confirmed that the multinucleation phenotype was really an increase in nucleus number *per* cell rather than just multilobular nuclei (data not shown). All multinucleated cells exhibited centrosome amplification. We also observed aberrant mitotic structures such as lagging chromosomes and DNA strands between dividing cells in cells overexpressing GFP-aurC-WT or GFP-aurC-CA (data not shown). Taken together, our results demonstrate that the overexpression of active Aurora kinase C induces centrosome amplification and polyploidy, defects frequently observed in cancers cells.

### Transformation of NIH-3T3 cells is correlated to the quantity of active kinase GFP-Aurora-C

**Figure 5:**
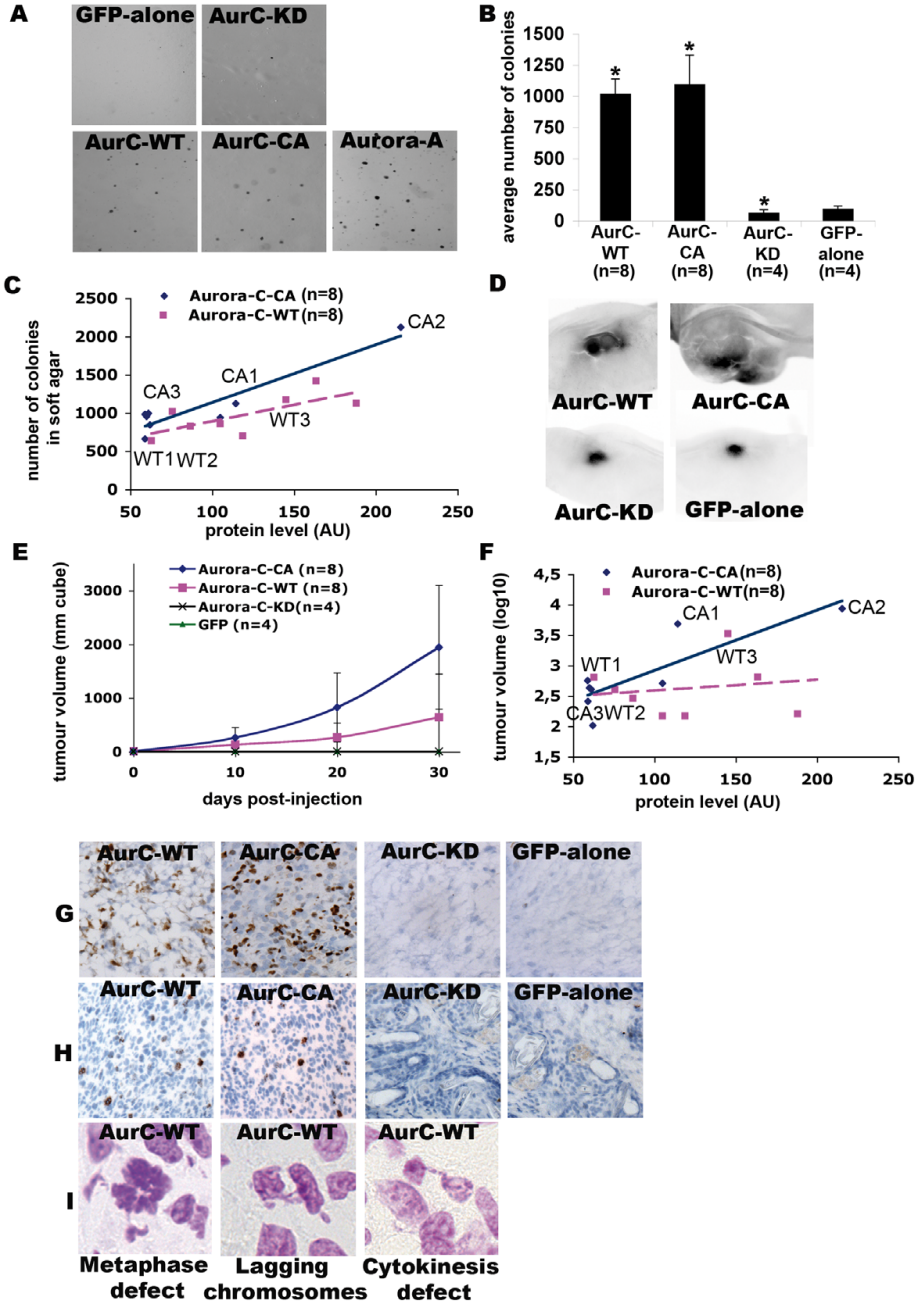
Cell growth in soft agar and tumour formation correlated with the quantity of active Aurora-C. (**A-C**) Stable cell clones of GFP-aurC-WT (n = 9), GFP-aurC-CA (n = 9), GFP-aurC-KD (n = 4), GFP-alone (n = 4) and GFP-AurA (n = 2) were tested in a soft agar growth assay. (**A**) Foci of colonies of GFP-aurC-WT, GFP-aurC-CA and GFP-aurA stable cell lines and very negligible number of very small colonies of GFP-aurC-KD and GFP-alone cell lines. (**B**) Histogram of the average number of colonies. We performed non-parametric Mann-Whitney test. Results were considered as statistically significant (*) for a p-value under 0.05 when compared to GFP-alone condition. (**C**) The dot blot shows a positive correlation between protein expression level of GFP-aurC-WT and GFP-aurC-CA, quantified for each clone on immunoblot by ImageJ, and the number of colonies in soft agar. Full line and dotted line are linear regression for GFP-AurC-CA and GFP-AurC-WT respectively. The label of the cell lines are named after in Figure 1C. (**D–I**) Female immunocompromized mice (SWISS, nu/nu) were injected subcutaneously in the abdomen. We injected GFP-aurC-WT (n = 8 clones), GFP-aurC-CA (n = 8), GFP-aurC-KD (n = 4), and GFP-alone (n = 4) cell lines. (**D**) Visualization of the tumours formed by injecting GFP-aurC-WT and GFP-aurC-CA stable cell lines, and the remaining injected cells of GFP-aurC-KD and GFP-alone on the day of sacrifice. For these images, the cell line injected were WT3, CA2, KD2 and GFP2 cell lines as named in Figure 1C. (**E**) The graph indicates an appearance and a gradual increase of tumour volume with GFP-aurC-WT and GFP-aurC-CA cell lines and no tumour formation in GFP-aurC-KD and GFP-alone cell lines. (**F**) The dot blot shows a positive correlation between expression level of GFP-aurC-WT and GFP-aurC-CA, quantified for each clone on immunoblot by ImageJ, and the tumour volume at day 30. Full line and dotted line are linear regression for GFP-AurC-CA and GFP-AurC-WT respectively. (**G–I**) Immunohistochemistry was performed on frozen sections of tumours or remaining injected cells with (**G**) rabbit monoclonal KI-67, a proliferation marker from late G1 to M-phase and (**H**) anti-phospho histone-H3 ser-10 and anti-HRP secondary antibodies, and (**I**) Feulgen staining.

Only transformed cells can grow on semi-liquid materials, like soft agar, as they lose the property of cell-to-cell contact inhibition and can grow over one another forming colonies. Overexpression of Aurora-A induces cell transformation [35], [36] while overexpression of Aurora-B cannot form colonies on soft agar [37]. Overexpressed Aurora-C behaves like Aurora-A in interphase and like Aurora-B in mitosis, does it posses any oncogenic activity? We assessed the potential of transformation of Aurora-C in soft agar assay using GFP-aurC-WT (n = 9), GFP-aurC-CA (n = 9), GFP-aurC-KD (n = 4) and GFP-alone (n = 4) NIH-3T3 stable cell clones **(** **Figure 5A, B** **)**. All the clones of GFP-aurC-WT or GFP-aurC-CA formed a large number of colonies. Similar results were obtained with GFP-aurA stable cell line used as a positive control for soft agar growth [18]. In contrast, stable cell clones of GFP-aurC-KD or GFP-alone formed negligible number of very small colonies. Interestingly, the higher the level of expression of active Aurora-C, the higher the colonies numbers it was **(** **Figure 5C** **)**.

To test whether NIH-3T3 cells overexpressing GFP-aurC were able to induce neoplastic transformation *in vivo*, clones overexpressing GFP-aurC-WT (n = 8), GFP-aurC-CA (n = 8), GFP-aurC-KD (n = 4), and GFP-alone (n = 4) were injected subcutaneously into immuno-compromised Swiss nu/nu mice **(** **Figure 4D, E** **)**. We observed that only the cells overexpressing GFP-aurC-WT, GFP-aurC-CA or aurC-WT gave rise to tumour formation within 30 days **(** **Figure 4E** and data not shown for aurC-WT). We never observed tumour with NIH3T3 cells overexpressing GFP-aurC-KD or GFP-alone. Here again, we were able to correlate tumour aggressiveness to high level of expression of active Aurora-C **(** **Figure 4F** **)**. These data are concordant with previous report showing that Aurora-C protein overexpression in cells derived from thyroid cancers correlated with the aggressiveness of the tumours [38].

We further analysed semi-quantitatively the proliferation status of the tumours obtained from GFP-aurC-WT and GFP-aurC-CA overexpressing cell lines. More than 50% and 40% of cells from respectively GFP-aurC-CA and GFP-aurC-WT tumours were positive for Ki-67 antigen, a marker of cycling cells, compared to less than 2% of GFP-aurC-KD and GFP-alone injected cells **(** **Figure 5G** **)**. More than 10% of cells overexpressing GFP-aurC-WT or GFP-aurC-CA were positive for histone H3-Serine10 phosphorylation, a M-phase marker, compared to less than 1% of cells overexpressing GFP-aurC-KD and GFP-alone **(** **Figure 5H** **)**. We found more than 90% of abnormal metaphase and more than 85% of both lagging chromosomes and cytokinesis defects in Feulgen staining of tumours induced by GFP-aurC-WT or GFP-aurC-CA **(** **Figure 5I** **)**. No such types of defects were observed in cells overexpressing GFP-aurC-KD or GFP-alone. Thus, the histological analysis of these tumours confirmed high proliferation rate and defects in mitosis of both GFP-aurC-WT and GFP-aurC-CA induced-tumors.

We concluded that only active GFP-aurC is oncogenic and induces tumour when it is overexpressed.

## Discussion

An overexpression of the three kinases of Aurora family has been detected in many human cancers [2]–[5]. In this report, we ask whether Aurora-C overexpression can result in cell transformation and tumour formation. We compared the potential to induce cell growth in soft agar and tumor of stable cell lines overexpressing GFP-aurC-WT, GFP-AurC-K72R (GFP-aurC-KD expressing kinase dead GFP-tagged aurC), GFP-aurC-T191D (GFP-aurC-CA expressing the constitutively active GFP-tagged aurC) and GFP as a control.

### Aurora-C-K72R kinase dead

In our hand, kinase dead mutant-K72R (GFP-AurC-KD or AurC-KD) did not induce centrosome amplification nor multinucleation in NIH-3T3 cells on the contrary to previous published data in other cell types [5], [27], [33], [39], [40]. Overexpression of GFP-AurC-KD did not displace either Aurora-B at centromeres (Figure 3). Taken together, our results suggest that Aurora-C kinase dead did not compete with endogenous Aurora-A or Aurora-B.

### Multinucleation and extra-centrosomes

We demonstrated that the overexpression of active Aurora kinase C induces centrosome amplification and multinucleation. All Aurora kinases are required for cell cycle regulation. Abnormal expression of Aurora kinases causes extra-centrosomes and multinucleation [5], [17]–[18], [26], [33], [36], [41], [53]. Inhibition or overexpression of Aurora-B results also in multinucleation [41]–[43]. Both Aurora-A and Aurora-B overexpression phenotypes are aggravated in the absence of active p53 [33], [41]. An elimination of the p53-dependent checkpoint may be evoked [44] to explain centrosome amplification and multinucleation induced by Aurora-C. Moreover, overexpressed Aurora-C kinase behaves like a dominant negative kinase for Aurora-B leading to cytokinesis defect that could explain the multinucleation phenotype observed in Aurora-C overexpressing cells [33].

### Aurora-C-T191D causes very aggressive tumors

It was reported that Aurora-C-T191D is a hyperactive mutant with a relative activity seven fold higher than the activity of Aurora-C-WT [25], [45]. In our hand in NIH-3T3 cells, GFP-aurC-T191D was constitutively active (GFP-AurC-CA) and its relative activity was very close to that of GFP-aurC-WT based on *in vitro* kinase assay (data not shown) and *in vivo* Histone H3 phosphorylation **(** **Figure 1E,F** **)**. The amplitude of centrosome amplification and multinucleation was similar with GFP-aurC-WT and GFP-aurC-CA **(** **Figure 2** **)**. In case of growth in soft agar and mouse tumour growth, however, clones from GFP-aurC-CA induced very aggressive tumours as compared to GFP-aurC-WT clones **(** **Figure 5A–E** **)**. These data underline that the intensity of kinase activity correlates with the aggressiveness of the tumours. That concept is worthy to be validated in human cancer, although a correlation has already been observed in thyroid cancers [38].

### Direct implication of Aurora-C in oncogenesis

Although all Aurora kinases are found overexpressed in cancer cells, their direct implication in oncogenesis varies. What would be the main difference between Aurora-B and Aurora-C that could explain the oncogenicity of Aurora-C? This might be linked to the different behaviour in interphase. In G2 phase cells, Aurora-B is found only in the nucleus whereas Aurora-C is cytoplasmic [33], [46]. During interphase Aurora-C localizes to the centrosomes just like Aurora-A, both of them demonstrating oncogenic potentials. Moreover, centrosome amplification, a common feature of Aurora-A and Aurora-C overexpression, is a frequent event in almost all types of solid cancers [47]–[52], [53]. Interestingly, the kinase activity of Aurora-A is not essential for induction of centrosome amplification, however, the oncogenic transformation requires kinase activity. Aurora-B by itself cannot induce transformation of cells but augments Ras-mediated transformation [37]. Aurora-B and -C have overlapping functions and compete each other for their substrates and other chromosome passenger proteins [5]. INCENP and Survivin have stronger affinity for Aurora-B than for Aurora-C [5] but interestingly Aurora-C can complement the functions of Aurora-B in mitotic cells. Whether the oncogenic activity of Aurora-C is related to its interphase function (Aurora-A like) or to its mitotic function related to its chromosome passenger behaviour (Aurora-B like) remains to be deciphered.

Two sequences in Aurora-A mediate its degradation at the end of mitosis: the destruction box (D-box at the carboxy-terminal domain) and the D-box activating domain (DAD, or A-box, at the amino-terminal domain). It is known that the A-box/DAD is absent from Aurora B and C, and their D-boxes are not targeted by the APC/C during mitotic exit [55]. Moreover, Aurora kinase-C is less susceptible than Aurora-B to degradation after anaphase by APC/Cdh1-mediated degradation [56]. The half-life of Aurora kinase-C needs to be deciphered throughout the cell cycle and the role of D-box should be adressed. According to Ulisse *et al*, the D-box present in the catalytic domain of Aurora-C may normalize the amount of protein *in vivo* [38].

It is likely that the presence of Aurora-C at the centrosome results in its oncogenic activity. We think, however, that the lack of A-box/DAD is not sole agent to be responsible for oncogenicity. Indeed, Aurora-A that has an active A-box/DAD domain is also an oncogene.

In conclusion, we show that overexpression of Aurora-C in somatic cells has an oncogenic potential that is dependent on its kinase activity. We also show that tumour aggressiveness is positively correlated to the quantity of active Aurora-C kinase. Together all these data makes Aurora-C kinase a novel excellent target of anticancer therapy.

### Comment

*While we were preparing this manuscript, an article demonstrating that AURKC enhances the transformation and tumourigenicity of epithelial cells was published* [54]. *Our results are in total agreement with these data. Taken together, our data and that article reinforce the active role of Aurora-C in tumourigenity, and place Aurora-C as a potential target of cancer therapies*.

## Materials and Methods

### Vectors

Human Aurora-C cDNA was obtained from pET21b-Aurora-C (Dutertre et al., 2005) and inserted into peEGFP-C3 plasmid (Clontech). This vector was called GFP-aurC-WT. The GFP-AurC-K72R (GFP-aurC-KD expressing kinase dead GFP-tagged aurC) and GFP-aurC-T191D (GFP-aurC-CA expressing the constitutively active GFP-tagged aurC) vectors were obtained by PCR site directed mutagenesis (Quick change site-directed mutagenesis kit, Stratagene) using GFP-aurC-WT plasmid as a template. The GFP-alone empty vector pEGFP-C3 was used as a negative control.

### Cell lines and transfection

NIH-3T3 cells (ATCC) were grown in Dulbecco's Modified-Eagle Medium (GIBCO) containing 10% fetal bovine serum (PAA) and 1% Penstrep (GIBCO). Cells were transfected in Jet Prime buffers (Polyplus) with GFP-aurC-WT, GFP-aurC-CA, GFP-aurC-KD and GFP-alone plasmids following manufacturer's instructions. For stable NIH-3T3 cell line establishment, 800 µg/ml Geneticin G-418 was added in culture media for 14 days, clones were selected and kept in 800 µg/ml Geneticin G-418.

### Western blotting

Proteins were extracted in RIPA buffer containing protease inhibitor cocktail (Roche). Western blots were run into 10% SDS PAGE gels, transferred and revealed using mouse Anti-GFP antibody (#1814460, 1∶1000, Sigma), polyclonal rabbit Anti-Aurora-C antibody (#38-9400, 1∶250, Zymed), and anti-beta tubulin antibody (T-4026-Sigma). In some experiments as stated in the figure legends, proteins extracts were immunoprecipitated with G-sepharose and mouse anti-GFP or polyclonal rabbit anti-Aurora-C antibodies according to manufacturer's procedure. Expression of Aurora-C protein were measured for anti-GFP western blot and analysed by ImageJ software.

### Immunofluorescence

Cells were fixed in cold methanol for 10 minutes at −20°C, washed and saturated with 1%BSA TBS. Primary antibodies in 1%BSA TBS were added on the cells (mouse anti-gamma tubulin, #GTU-88- T6557, 1∶2500, Sigma; rabbit anti-phospho histone H3 ser-10- #06570, 1∶1000, Millipore; rabbit anti-GFP- #632375, 1∶2000, Clontech) for 2 hours at 4°C, washed and incubated with secondary antibodies (anti-mouse Alexa-555, 1∶1000; anti-rabbit Alexa-555, 1∶1000; anti-rabbit Alexa-488, 1∶1000, Invitrogen) for 1 hour at room temperature, washed and mounted in Prolong-Gold (Invitrogen) containing DAPI, a DNA staining dye. Images were collected using Leica DMRXA2 fluorescent microscope. Photographs were taken using a black and white cool snap ES camera (Roper Scientific). Images were processed using Metamorph Software (Universal Imaging) and ImageJ for quantification of H3 staining intensity. All the images have been taken for quantitative analysis in ImageJ with the same settings for image acquisition (intensity, exposure time, magnification).

### Soft Agar Assay

Stable cell clones of GFP-aurC-WT (n = 9), GFP-aurC-CA (n = 9), GFP-aurC-KD (n = 4), GFP-alone (n = 4) and GFP-AurA (n = 2) were tested in a soft agar growth assay. Ten thousands cells/well in a 6-well plate in triplicate were grown in 2 ml top agar containing 2× DMEM media, 20% fetal bovine serum and 1% agarose. Geneticin-G-418 was added 24 hours after seeding. Media were changed twice a week. Thirty days after seeding, well plates were stained with 0.005% crystal violet dye and the numbers of colonies were counted.

### Ethics

Experiments with mice were conducted in accordance with University of Rennes 1 and French Ministère de l'Enseignement Supérieur et de la Recherche authorization (agreement #5346 to C. Prigent) and guidelines from the “Direction Départementale des services Vétérinaires d' Ille et Vilaine”.

### Tumour growth

Female immunocompromized mice (SWISS, nu/nu) of 3 weeks of age were housed in microisolator units under controlled humidity and temperature. They were fed with sterile diet and water. Stable cell clones were stained overnight with 10 µg/ml DilC18(3) (FluoProbes) prior to injection. Seven millions cells of each clone were injected subcutaneously in the abdomen. We injected GFP-aurC-WT (n = 8 clones), GFP-aurC-CA (n = 8), GFP-aurC-KD (n = 4), and GFP-alone (n = 4) cell lines. Each mouse was injected with two different clones, one on each side of the abdomen. Tumour sizes were monitored every 10 days by direct measurement with vernier caliper and, on the day of sacrifice, using Kodak image station 2000 by an excitation of 535 nm that detected cells stained with DilC18(3). Tumour volumes were calculated according to the formula, LxWxHxπ/6. Mice were sacrificed when tumours reached a size of about 1 cm or after 7–8 weeks of monitoring. Tumours were removed, immediately frozen in liquid nitrogen and then stored at −80°C for further analyses.

### Immunohistochemistry

Ten micrometers thick frozen sections of tumours or remaining injected cells were cut on a cryostat (Leica, Milton Keynes, UK) and mounted onto uncoated glass slides. Immuno-histochemistry was performed with rabbit monoclonal KI-67, a proliferation marker from late G1 to M-phase (1∶200, Epitomics, clone SP6) and anti-phospho histone-H3 ser-10 (Millipore) and anti-HRP (Jackson) secondary antibodies. We also performed Feulgen staining.

### Statistics

We performed non-parametric Mann-Whitney test. Results were considered as statistically significant (*) for a p-value under 0.05 when compared to GFP-alone condition.
